# Benefits and risks of noninvasive oxygenation strategy in COVID-19: a multicenter, prospective cohort study (COVID-ICU) in 137 hospitals

**DOI:** 10.1186/s13054-021-03784-2

**Published:** 2021-12-08

**Authors:** COVID-ICU group, for the REVA network, COVID-ICU investigators, Matthieu Schmidt, Alexandre Demoule, David Hajage, Tài Pham, Alain Combes, Martin Dres, Said Lebbah, Antoine Kimmoun, Alain Mercat, Gaëtan Beduneau, Jessica Palmyre, Margot Prevost, Jean-Damien Ricard, Alexis Ferré, Pierre-Marie Fayolle, Christophe Girault, Gael Pradel, Alain Mercat, Pierre Asfar, François Beloncle, Julien Demiselle, Tài Pham, Arthur Pavot, Xavier Monnet, Christian Richard, Alexandre Demoule, Martin Dres, Julien Mayaux, Alexandra Beurton, Cédric Daubin, Richard Descamps, Aurélie Joret, Damien Du Cheyron, Frédéric Pene, Jean-Daniel Chiche, Mathieu Jozwiak, Paul Jaubert, Guillaume Voiriot, Muriel Fartoukh, Marion Teulier, Clarisse Blayau, Erwen L’Her, Cécile Aubron, Laetitia Bodenes, Nicolas Ferriere, Johann Auchabie, Anthony Le Meur, Sylvain Pignal, Thierry Mazzoni, Jean-Pierre Quenot, Pascal Andreu, Jean-Baptiste Roudau, Marie Labruyère, Saad Nseir, Sébastien Preau, Julien Poissy, Daniel Mathieu, Sarah Benhamida, Rémi Paulet, Nicolas Roucaud, Martial Thyrault, Florence Daviet, Sami Hraiech, Gabriel Parzy, Aude Sylvestre, Sébastien Jochmans, Anne-Laure Bouilland, Mehran Monchi, Marc Danguy des Déserts, Quentin Mathais, Gwendoline Rager, Pierre Pasquier, Reignier Jean, Seguin Amélie, Garret Charlotte, Canet Emmanuel, Jean Dellamonica, Clément Saccheri, Romain Lombardi, Yanis Kouchit, Sophie Jacquier, Armelle Mathonnet, Mai-Ahn Nay, Isabelle Runge, Frédéric Martino, Laure Flurin, Amélie Rolle, Michel Carles, Rémi Coudroy, Arnaud W. Thille, Jean-Pierre Frat, Maeva Rodriguez, Pascal Beuret, Audrey Tientcheu, Arthur Vincent, Florian Michelin, Fabienne Tamion, Dorothée Carpentier, Déborah Boyer, Christophe Girault, Valérie Gissot, Stéphan Ehrmann, Charlotte Salmon Gandonniere, Djlali Elaroussi, Agathe Delbove, Yannick Fedun, Julien Huntzinger, Eddy Lebas, Grâce Kisoka, Céline Grégoire, Stella Marchetta, Bernard Lambermont, Laurent Argaud, Thomas Baudry, Pierre-Jean Bertrand, Auguste Dargent, Christophe Guitton, Nicolas Chudeau, Mickaël Landais, Cédric Darreau, Alexis Ferre, Antoine Gros, Guillaume Lacave, Fabrice Bruneel, Mathilde Neuville, Jérôme Devaquet, Guillaume Tachon, Richard Gallot, Riad Chelha, Arnaud Galbois, Anne Jallot, Ludivine Chalumeau Lemoine, Khaldoun Kuteifan, Valentin Pointurier, Louise-Marie Jandeaux, Joy Mootien, Charles Damoisel, Benjamin Sztrymf, Matthieu Schmidt, Alain Combes, Juliette Chommeloux, Charles Edouard Luyt, Frédérique Schortgen, Leon Rusel, Camille Jung, Florent Gobert, Damien Vimpere, Lionel Lamhaut, Bertrand Sauneuf, Liliane Charrrier, Julien Calus, Isabelle Desmeules, Benoît Painvin, Jean-Marc Tadie, Vincent Castelain, Baptiste Michard, Jean-Etienne Herbrecht, Mathieu Baldacini, Nicolas Weiss, Sophie Demeret, Clémence Marois, Benjamin Rohaut, Pierre-Henri Moury, Anne-Charlotte Savida, Emmanuel Couadau, Mathieu Série, Nica Alexandru, Cédric Bruel, Candice Fontaine, Sonia Garrigou, Juliette Courtiade Mahler, Maxime Leclerc, Michel Ramakers, Pierre Garçon, Nicole Massou, Ly Van Vong, Juliane Sen, Nolwenn Lucas, Franck Chemouni, Annabelle Stoclin, Alexandre Avenel, Henri Faure, Angélie Gentilhomme, Sylvie Ricome, Paul Abraham, Céline Monard, Julien Textoris, Thomas Rimmele, Florent Montini, Gabriel Lejour, Thierry Lazard, Isabelle Etienney, Younes Kerroumi, Claire Dupuis, Marine Bereiziat, Elisabeth Coupez, François Thouy, Clément Hoffmann, Nicolas Donat, Anne Chrisment, Rose-Marie Blot, Antoine Kimmoun, Audrey Jacquot, Matthieu Mattei, Bruno Levy, Ramin Ravan, Loïc Dopeux, Jean-Mathias Liteaudon, Delphine Roux, Brice Rey, Radu Anghel, Deborah Schenesse, Vincent Gevrey, Jermy Castanera, Philippe Petua, Benjamin Madeux, Otto Hartman, Michael Piagnerelli, Anne Joosten, Cinderella Noel, Patrick Biston, Thibaut Noel, Gurvan L. E. Bouar, Messabi Boukhanza, Elsa Demarest, Marie-France Bajolet, Nathanaël Charrier, Audrey Quenet, Cécile Zylberfajn, Nicolas Dufour, Buno Mégarbane, Sqébastian Voicu, Nicolas Deye, Isabelle Malissin, François Legay, Matthieu Debarre, Nicolas Barbarot, Pierre Fillatre, Bertrand Delord, Thomas Laterrade, Tahar Saghi, Wilfried Pujol, Pierre Julien Cungi, Pierre Esnault, Mickael Cardinale, Vivien Hong Tuan Ha, Grégory Fleury, Marie-Ange Brou, Daniel Zafimahazo, David Tran-Van, Patrick Avargues, Lisa Carenco, Nicolas Robin, Alexandre Ouali, Lucie Houdou, Christophe Le Terrier, Noémie Suh, Steve Primmaz, Jérome Pugin, Emmanuel Weiss, Tobias Gauss, Jean-Denis Moyer, Catherine Paugam Burtz, Béatrice La Combe, Rolland Smonig, Jade Violleau, Pauline Cailliez, Jonathan Chelly, Antoine Marchalot, Cécile Saladin, Christelle Bigot, Pierre-Marie Fayolle, Jules Fatséas, Amr Ibrahim, Dabor Resiere, Rabih Hage, Clémentine Cholet, Marie Cantier, Pierre Trouiler, Philippe Montravers, Brice Lortat-Jacob, Sebastien Tanaka, Alexy Tran Dinh, Jacques Duranteau, Anatole Harrois, Guillaume Dubreuil, Marie Werner, Anne Godier, Sophie Hamada, Diane Zlotnik, Hélène Nougue, Armand Mekontso-Dessap, Guillaume Carteaux, Keyvan Razazi, Nicolas De Prost, Nicolas Mongardon, Olivier Langeron, Eric Levesque, Arié Attias, Charles de Roquetaillade, Benjamin G. Chousterman, Alexandre Mebazaa, Etienne Gayat, Marc Garnier, Emmanuel Pardo, Lea Satre-Buisson, Christophe Gutton, Elise Yvin, Clémence Marcault, Elie Azoulay, Michael Darmon, Hafid Ait Oufella, Geoffroy Hariri, Tomas Urbina, Sandie Mazerand, Nicholas Heming, Francesca Santi, Pierre Moine, Djillali Annane, Adrien Bouglé, Edris Omar, Aymeric Lancelot, Emmanuelle Begot, Gaétan Plantefeve, Damien Contou, Hervé Mentec, Olivier Pajot, Stanislas Faguer, Olivier Cointault, Laurence Lavayssiere, Marie-Béatrice Nogier, Matthieu Jamme, Claire Pichereau, Jan Hayon, Hervé Outin, François Dépret, Maxime Coutrot, Maité Chaussard, Lucie Guillemet, Pierre Goffin, Romain Thouny, Julien Guntz, Laurent Jadot, Romain Persichini, Vanessa Jean-Michel, Hugues Georges, Thomas Caulier, Gaël Pradel, Marie-Hélène Hausermann, Thi My Hue Nguyen-Valat, Michel Boudinaud, Emmanuel Vivier, Sylvène Rosseli, Gaël Bourdin, Christian Pommier, Marc Vinclair, Simon Poignant, Sandrine Mons, Wulfran Bougouin, Franklin Bruna, Quentin Maestraggi, Christian Roth, Laurent Bitker, François Dhelft, Justine Bonnet-Chateau, Mathilde Filippelli, Tristan Morichau-Beauchant, Stéphane Thierry, Charlotte Le Roy, Mélanie Saint Jouan, Bruno Goncalves, Aurélien Mazeraud, Matthieu Daniel, Tarek Sharshar, Cyril Cadoz, Rostane Gaci, Sébastien Gette, Guillaune Louis, Sophe-Caroline Sacleux, Marie-Amélie Ordan, Aurélie Cravoisy, Marie Conrad, Guilhem Courte, Sébastien Gibot, Younès Benzidi, Claudia Casella, Laurent Serpin, Jean-Lou Setti, Marie-Catherine Besse, Anna Bourreau, Jérôme Pillot, Caroline Rivera, Camille Vinclair, Marie-Aline Robaux, Chloé Achino, Marie-Charlotte Delignette, Tessa Mazard, Frédéric Aubrun, Bruno Bouchet, Aurélien Frérou, Laura Muller, Charlotte Quentin, Samuel Degoul, Xavier Stihle, Claude Sumian, Nicoletta Bergero, Bernard Lanaspre, Hervé Quintard, Eve Marie Maiziere, Pierre-Yves Egreteau, Guillaume Leloup, Florin Berteau, Marjolaine Cottrel, Marie Bouteloup, Matthieu Jeannot, Quentin Blanc, Julien Saison, Isabelle Geneau, Romaric Grenot, Abdel Ouchike, Pascal Hazera, Anne-Lyse Masse, Suela Demiri, Corinne Vezinet, Elodie Baron, Deborah Benchetrit, Antoine Monsel, Grégoire Trebbia, Emmanuelle Schaack, Raphaël Lepecq, Mathieu Bobet, Christophe Vinsonneau, Thibault Dekeyser, Quentin Delforge, Imen Rahmani, Bérengère Vivet, Jonathan Paillot, Lucie Hierle, Claire Chaignat, Sarah Valette, Benoït Her, Jennifier Brunet, Mathieu Page, Fabienne Boiste, Anthony Collin, Florent Bavozet, Aude Garin, Mohamed Dlala, Kais Mhamdi, Bassem Beilouny, Alexandra Lavalard, Severine Perez, Benoit Veber, Pierre-Gildas Guitard, Philippe Gouin, Anna Lamacz, Fabienne Plouvier, Bertrand P. Delaborde, Aïssa Kherchache, Amina Chaalal, Jean-Damien Ricard, Marc Amouretti, Santiago Freita-Ramos, Damien Roux, Jean-Michel Constantin, Mona Assefi, Marine Lecore, Agathe Selves, Florian Prevost, Christian Lamer, Ruiying Shi, Lyes Knani, Sébastien Pili Floury, Lucie Vettoretti, Michael Levy, Lucile Marsac, Stéphane Dauger, Sophie Guilmin-Crépon, Hadrien Winiszewski, Gael Piton, Thibaud Soumagne, Gilles Capellier, Jean-Baptiste Putegnat, Frédérique Bayle, Maya Perrou, Ghyslaine Thao, Guillaume Géri, Cyril Charron, Xavier Repessé, Antoine Vieillard-Baron, Mathieu Guilbart, Pierre-Alexandre Roger, Sébastien Hinard, Pierre-Yves Macq, Kevin Chaulier, Sylvie Goutte, Patrick Chillet, Anaïs Pitta, Barbara Darjent, Amandine Bruneau, Sigismond Lasocki, Maxime Leger, Soizic Gergaud, Pierre Lemarie, Nicolas Terzi, Carole Schwebel, Anaïs Dartevel, Louis-Marie Galerneau, Jean-Luc Diehl, Caroline Hauw-Berlemont, Nicolas Péron, Emmanuel Guérot, Abolfazl Mohebbi Amoli, Michel Benhamou, Jean-Pierre Deyme, Olivier Andremont, Diane Lena, Julien Cady, Arnaud Causeret, Arnaud De La Chapelle, Christophe Cracco, Stéphane Rouleau, David Schnell, Camille Foucault, Cécile Lory, Thibault Chapelle, Vincent Bruckert, Julie Garcia, Abdlazize Sahraoui, Nathalie Abbosh, Caroline Bornstain, Pierre Pernet, Florent Poirson, Ahmed Pasem, Philippe Karoubi, Virginie Poupinel, Caroline Gauthier, François Bouniol, Philippe Feuchere, Florent Bavozet, Anne Heron, Serge Carreira, Malo Emery, Anne Sophie Le Floch, Luana Giovannangeli, Nicolas Herzog, Christophe Giacardi, Thibaut Baudic, Chloé Thill, Said Lebbah, Jessica Palmyre, Florence Tubach, David Hajage, Nicolas Bonnet, Nathan Ebstein, Stéphane Gaudry, Yves Cohen, Julie Noublanche, Olivier Lesieur, Arnaud Sément, Isabel Roca-Cerezo, Michel Pascal, Nesrine Sma, Gwenhaël Colin, Jean-Claude Lacherade, Gauthier Bionz, Natacha Maquigneau, Pierre Bouzat, Michel Durand, Marie-Christine Hérault, Jean-Francois Payen

**Affiliations:** Service de Médecine Intensive – Réanimation, Hôpital Pitié-Salpêtrière, 83 Boulevard de l’Hôpital, 75013 Paris, France

**Keywords:** Acute respiratory distress syndrome, Mechanical ventilation, COVID-19, Outcome, High-flow nasal cannula, Intubation, Mortality, Acute respiratory failure

## Abstract

**Rational:**

To evaluate the respective impact of standard oxygen, high-flow nasal cannula (HFNC) and noninvasive ventilation (NIV) on oxygenation failure rate and mortality in COVID-19 patients admitted to intensive care units (ICUs).

**Methods:**

Multicenter, prospective cohort study (COVID-ICU) in 137 hospitals in France, Belgium, and Switzerland. Demographic, clinical, respiratory support, oxygenation failure, and survival data were collected. Oxygenation failure was defined as either intubation or death in the ICU without intubation. Variables independently associated with oxygenation failure and Day-90 mortality were assessed using multivariate logistic regression.

**Results:**

From February 25 to May 4, 2020, 4754 patients were admitted in ICU. Of these, 1491 patients were not intubated on the day of ICU admission and received standard oxygen therapy (51%), HFNC (38%), or NIV (11%) (*P* < 0.001). Oxygenation failure occurred in 739 (50%) patients (678 intubation and 61 death). For standard oxygen, HFNC, and NIV, oxygenation failure rate was 49%, 48%, and 60% (*P* < 0.001). By multivariate analysis, HFNC (odds ratio [OR] 0.60, 95% confidence interval [CI] 0.36–0.99, *P* = 0.013) but not NIV (OR 1.57, 95% CI 0.78–3.21) was associated with a reduction in oxygenation failure). Overall 90-day mortality was 21%. By multivariable analysis, HFNC was not associated with a change in mortality (OR 0.90, 95% CI 0.61–1.33), while NIV was associated with increased mortality (OR 2.75, 95% CI 1.79–4.21, *P* < 0.001).

**Conclusion:**

In patients with COVID-19, HFNC was associated with a reduction in oxygenation failure without improvement in 90-day mortality, whereas NIV was associated with a higher mortality in these patients. Randomized controlled trials are needed.

**Supplementary Information:**

The online version contains supplementary material available at 10.1186/s13054-021-03784-2.

## Introduction

Severe acute respiratory syndrome coronavirus 2 (SARS-CoV-2) is the causative agent of the ongoing coronavirus disease 2019 (COVID-19) pandemic. Understanding how clinicians can address the demand for emergency mass critical care and fine-tune the standard of care regarding oxygenation management is of the utmost importance. For instance, guidance about how to allocate scarce critical care resources such as ventilators should be made using data showing that safe alternatives to standard oxygen therapy can avoid intubation without lessening survival probabilities.

In patients with de novo acute respiratory failure admitted to the intensive care unit (ICU), high-flow nasal cannula oxygen (HFNC) and noninvasive mechanical ventilation (NIV) improve oxygenation and reduce inspiratory effort and the work of breathing [1–3]. High-flow nasal cannula oxygen has shown clinical benefit by reducing the intubation rate [4], and its use is now recommended in de novo acute respiratory failure [5]. Noninvasive ventilation decreases the intubation rate [6], but NIV failure and subsequent intubation is associated with higher mortality compared to first-line intubation [7], and NIV is not recommended in de novo acute respiratory failure [6].

In COVID-19 patients, recent data suggest that HFNC and NIV are associated with a reduction in intubation rate, but without a clear benefit on mortality [8, 9].

We hypothesized that HFNC and NIV could be beneficial to the outcome of COVID-19 patients admitted to the ICU for acute respiratory failure. This study comprised a secondary analysis of the large international COVID-ICU study [10] with the following specific objectives: 1) to quantify the respective use of standard oxygen, HFNC, and NIV; 2) to determine oxygenation failure rate (i.e., intubation rate or death in the ICU without intubation) of standard oxygen, HFNC, and NIV and to evaluate the impact of HFNC and NIV on oxygen failure; and 3) to evaluate whether HFNC and NIV use is associated with a reduction in mortality.

## Methods

### Study design, patients

COVID-ICU is a multicenter, prospective cohort study conducted in 149 ICUs from 137 centers, across three countries (France, Switzerland, and Belgium). The main characteristics of these ICUs have been described elsewhere [10]. Centers were invited to participate by public announcements and through the REVA network (70 centers were active members of this network). COVID-ICU received approval from the ethical committee of the French Intensive Care Society (CE-SRLF 20–23) in accordance with local regulations. All patients or close relatives were informed that their data were included in the COVID-ICU cohort.

All consecutive patients over 16 years of age admitted to participating ICUs between February 25, 2020, and May 4, 2020, with laboratory-confirmed SARS-CoV-2 infection, were included. Laboratory confirmation for SARS-Cov-2 was defined as a positive result of real-time reverse transcriptase-polymerase chain reaction (RT-PCR) assay from either nasal or pharyngeal swabs, or lower respiratory tract aspirate. For the purposes of the present post hoc analysis, only patients who were not intubated on the day of ICU admission were included.

### Data collection

Day 1 was defined as the first day that the patient was in the ICU at 10 am. Each day, the study investigators completed a standardized electronic case report form. Baseline information collected at ICU admission was age, sex, body mass index, active smoking, Simplified Acute Physiology Score II score [11], Sequential Organ Failure Assessment [12], comorbidities, immunodeficiency (if present), clinical frailty scale [13], date of the first symptom, date of hospital, and ICU admissions. The case report form prompted investigators to provide a daily expanded data set including the use of respiratory support devices (standard oxygen, HFNC, or NIV), the need for invasive mechanical ventilation, the fraction of inspired oxygen (FiO_2_), blood gases at Day 1 (PaO_2_/FiO_2_ was calculated in all patients by converting O_2_ flow to estimated FiO_2_, see Additional file 1: Table S1), and standard laboratory parameters. Some patients received more than one of the three oxygenation techniques, in which case the most invasive was retained for further analyses, assuming NIV to be more invasive than HFNC and HFNC to be more invasive than standard oxygen.

Patient outcomes included the date of invasive mechanical ventilation, the date of ICU and hospital discharge, and vital status at ICU discharge, hospital discharge, and 28, 60, and 90 days after ICU admission. Patients who received invasive mechanical ventilation or who died before ICU discharge without being intubated were classified as oxygenation strategy failure. Patients treated with an oxygenation technique until assistance was no longer required were classified as success.

### Statistical analyses

Characteristics of patients were described as frequencies and percentages for categorical variables, and as means and standard deviations or medians and interquartile ranges for continuous variables. Categorical variables were compared by chi-square or Fisher's exact test, and continuous variables were compared by Student's *t* test or Wilcoxon's rank-sum test. Kaplan–Meier overall survival curves until Day 90 were computed and were compared using log-rank tests.

Variables associated with oxygenation failure, defined as intubation or death in the ICU without intubation, were assessed using multivariate logistic regression analyses, and the results are given as odds ratio (OR) with their 95% confidence interval (CI).

Baseline risk factors of death at Day 90 were assessed within the whole cohort using univariate and multivariate cox regression. Baseline variables (i.e., obtained during the first 24 h in the ICU) included in the multivariate model were defined a priori, and no variable selection was performed. A sensitivity analysis with multiple imputations was also performed.

A *P* value < 0.05 was considered statistically significant. Statistical analyses were conducted with R v3.5.1.

## Results

### Study population and oxygenation and ventilation modalities

Over the 10-week study period, 4754 patients were enrolled, of them 510 were lost to follow-up at Day 90. Vital status at Day 90 was available for the remaining 4244 patients. Data on initial oxygenation strategy were missing in 118 patients.

**Fig. 1:**
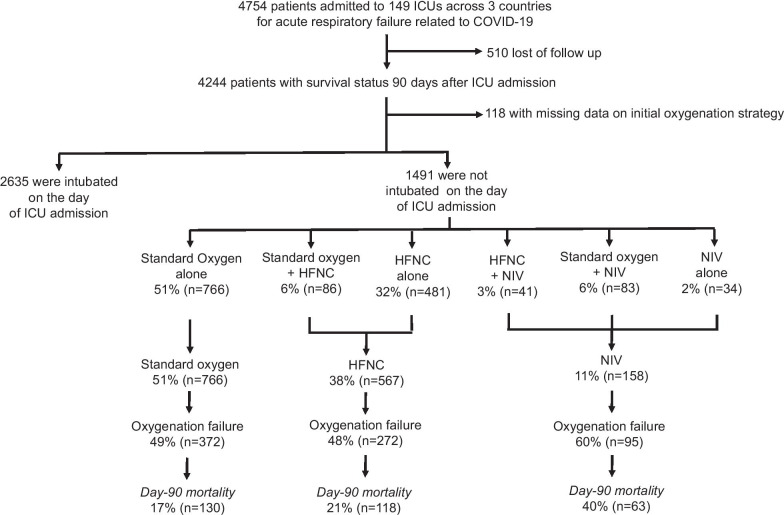
Flow of patient screening and inclusion. COVID-19, coronavirus disease 2019; ICU, intensive care unit; HFNC, high-flow nasal cannula; NIV, noninvasive ventilation

Figure 1 depicts the oxygenation and ventilation modalities following ICU admission. A total of 2635 patients were intubated on the day of ICU admission (time from ICU admission to intubation 2.0 [0.5–7.2] hours) and were excluded from the present analysis. The remaining 1491 patients were not intubated on the day of ICU admission.

Table 1 shows the demographic, clinical, and biological characteristics of the 1491 patients who were not intubated on ICU admission, overall and for each oxygenation strategy. Standard oxygen therapy, HFNC, and NIV were applied to 766 (51%), 567 (38%), and 158 (11%) patients, respectively. Among patients who received NIV, 11 (1%) received continuous positive airway pressure and the remaining 147 (10%) patients received pressure support ventilation. Noninvasive ventilation was exclusively delivered with a bucconasal or face facemask; helmet was not used. Patients who received either HFNC or NIV were older, had a higher SOFA score, were more severely hypoxemic, and were admitted to the ICU a longer period of time after first symptoms. The proportion of patients who received HFNC and NIV was higher between March 29 and May 4, 2020, than between February 25 and March 28, 2020. In patients receiving standard oxygen, oxygen flow was 6 (4–10) L/min. In those receiving HFNC, flow was 50 (40–60) L/min and FiO_2_ was 70 (60–90)%. In those receiving NIV, pressure support level was 8 (6–10) cmH_2_O, positive end-expiratory pressure was 7 (6–8) cmH_2_O and FiO_2_ was 60 (50–80)%. Overall, a do-not-intubate decision had been taken in 155 (10%) patients. This proportion was higher in patients who received NIV (23%, *n* = 36) than in those who received HFNC (10%, *n* = 54) or standard oxygen (8%, *n* = 65, *P* < 0.001).

**Table 1:** Demographic, clinical, and biological characteristics of 1491 patients not intubated on the day of intensive care unit (ICU) admission, according to oxygenation strategy

	No	All patients	Standard oxygen	HFNC	NIV	*P* value
		(*n* = 1491)	(*n* = 766)	(*n* = 567)	(*n* = 158)	
Age, years	1491	63 (54–71)	61 (53–70)	64 (55–72)	65 (56–72)	0.005
Women, *n *(%)	1488	397 (27)	212 (28)	140 (25)	45 (29)	0.389
Body mass index, kg/m^2^	1370	28 (25–31)	28 (25–32)	28 (25–31)	29 (26–33)	0.334
≥ 30 kg/m^2^, *n *(%)	1370	504 (37)	260 (37)	179 (35)	65 (45)	0.085
Active smokers, *n *(%)	1465	67 (5)	35 (5)	19 (4)	13 (8)	0.037
SAPS II score	1398	30 (23–37)	27 (21–35)	32 (27–39)	33 (26–41)	< 0.001
SOFA score at ICU admission	1290	3 (2–4)	2 (2–4)	3 (2–4)	3 (2–5)	< 0.001
Treated hypertension, *n *(%)	1485	687 (46)	331 (43)	263 (47)	93 (59)	0.001
Known diabetes, *n *(%)	1486	409 (28)	206 (27)	145 (26)	58 (37)	0.018
Immunodeficiency^a^, *n *(%)	1479	102 (7)	42 (6)	48 (9)	12 (8)	0.090
Clinical frailty scale	1379	2 (2–3)	2 (1–3)	2 (2–3)	2 (2–4)	< 0.001
Time between first symptoms and ICU admission, *days*	1435	9 (6–12)	8 (6–11)	9 (7–12)	9 (7–12)	< 0.001
**During the first 24 h in ICU**						
Hemodynamic component of the SOFA	1435	0 (0–0)	0 (0–0)	0 (0–0)	0 (0–0)	0.351
Renal component of the SOFA	1416	0 (0–0)	0 (0–0)	0 (0–0)	0 (0–0)	0.047
Corticosteroids^b^, *n *(%)	1476	165 (11)	50 (7)	85 (15)	30 (19)	< 0.001
**Blood gases**						
pH	1365	7.46 (7.42–7.48)	7.45 (7.42–7.48)	7.46 (7.44–7.49)	7.44 (7.41–7.48)	< 0.001
PaCO_2_, mmHg	1367	36 (32–40)	36 (33–40)	36 (32–39)	36 (32–43)	0.039
PaO_2_/FiO_2_^c^	1216	122 (82–177)	154 (89–219)	104 (77–134)	128 (86–169)	< 0.001
HCO_3_, mmol/L	1357	25 (23–27)	25 (23–27)	25 (23–27)	25 (23–28)	0.769
Lactate, mmol/L	1281	1.1 (0.9–1.5)	1.1 (0.8–1.5)	1.2 (0.9–1.5)	1.1 (0.9–1.5)	0.039
**Biology**						
Lymphocyte count, × 10^9^/L	1199	0.8 (0.6–1.2)	0.9 (0.6–1.2)	0.8 (0.6–1.1)	0.8 (0.6–1.1)	0.016
Platelet count, × 10^9^/L	1342	222 (167–289)	205 (159–275)	236 (177–302)	241 (177–294)	< 0.001
Total bilirubin, µmol/L	1023	9 (7–12)	9 (7–12)	9 (7–12)	9 (7–13)	0.085
Serum creatinine, µmol/L	1369	71 (59–94)	72 (60–91)	71 (57–92)	74 (59–110)	0.098
D–dimers, µg/L	548	1159 (647–2204)	1080 (675–2000)	1282 (708–2930)	1040 (538–1939)	0.097
Period of admission	1491					< 0.001
February 25 to March 28, 2020, *n *(%)		713 (48)	479 (63)	187 (33)	47 (30)	
March 29 to May 4, 2020, *n *(%)		778 (52)	287 (37)	380 (67)	111 (70)	
**Do-not-intubate, *n *(%)**	1491	155 (10%)	65 (8%)	54 (10%)	36 (23%)	< 0.001

Results are expressed as *n* (%) or median (25th–75th percentiles)

*HFNC* high-flow nasal cannula, *NIV* noninvasive ventilation, *SAPS* simplified acute physiology score, *SOFA* sequential organ failure assessment, *PaCO*_*2*_ partial pressure of carbon dioxide, *PaO*_*2*_*/FiO*_*2*_ partial pressure of oxygen to fraction of inspired oxygen ratio, *HCO3* bicarbonate

^a^Defined as hematological malignancies, active solid tumor, or having received specific anti-tumor treatment within a year, solid organ transplant, human immunodeficiency virus, or immunosuppressants

^b^Irrespective of the dose and the indication

^c^Calculated for all patients, including those on oxygen therapy by using conversion tables provided in the online supplement

### Factors associated with intubation and impact of HFNC and NIV on intubation

Invasive mechanical ventilation was started in 678 patients, and 61 patients died in the ICU without being intubated. These 739 (50%) patients were classified as oxygenation failure. The remaining 752 (50%) patients were classified as oxygenation success.

**Fig. 2:**
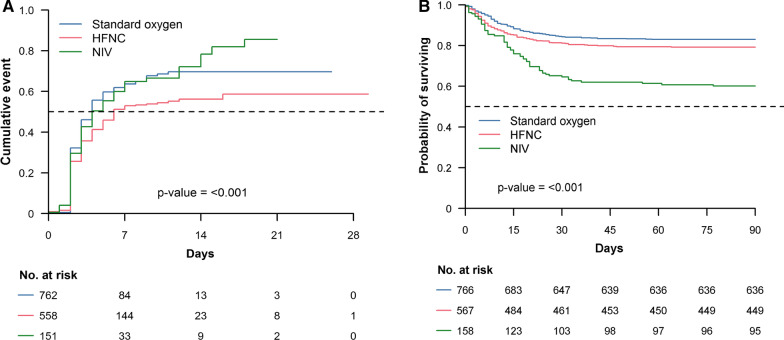
Left panel: Cumulative incidence of oxygenation failure according to the oxygenation technique in patients not intubated on the day of intensive care unit admission. Right panel: Cumulative incidence of hospital mortality according to the oxygenation technique. HFNC, high-flow nasal cannula; NIV, noninvasive ventilation;

Table 2 shows the intubation rate, mortality, and length of stay in patients who were not intubated on the day of admission. Intubation rate was not different between patients treated with standard oxygen, HFNC, and NIV. Intubation occurred after a longer period of time in patients treated with HFNC and NIV than in those treated with standard oxygen (Table 2). The proportion of patients who died without being intubated as well as the oxygenation failure rate were higher in the NIV group compared to those who received HFNC or standard oxygen. Additional file 1: Table S1 shows the univariate analysis of factors associated with oxygenation failure in patients who were not intubated on ICU admission. Variables associated with oxygenation failure by multivariable analysis were: severity as assessed by the SAPS II, time between the first symptoms and ICU admission, oxygenation technique, higher renal component of the SOFA score, lower PaO_2_/FiO_2_, higher blood lactate, and admission period after March 29, 2021 (Table 3). HFNC (OR 0.60, 95% CI 0.36–0.99, *P* = 0.013) but not NIV (OR 1.57, 95% CI 0.78–3.21) was associated with a reduction in oxygenation failure. The cumulative incidence of oxygenation failure for standard oxygen, HFNC, and NIV is shown in Fig. 2, Panel A.

**Table 2:** Outcome of 1491 patients not intubated on the day of intensive care unit (ICU) admission, according to oxygenation strategy

	No	All patients	Standard oxygen	HFNC	NIV	*P* value
		(*n* = 1491)	(*n* = 766)	(*n* = 567)	(*n* = 158)	
Invasive mechanical ventilation, *n *(%)	1491	678 (45)	359 (47)	242 (43)	77 (49)	0.217
Time between ICU admission and invasive mechanical ventilation, days	635	2 (1–3)	2 (1–2)	2 (1–3)	2 (1–3)	0.012
ICU mortality without being intubated, *n *(%)	1491	61 (4)	13 (2)	30 (5)	18 (11)	< 0.001
Oxygenation failure rate, *n *(%)	1491	739 (50)	372 (49)	272 (48)	95 (60)	< 0.001
ICU mortality, *n *(%)	1487	269 (18)	108 (14)	109 (19)	52 (33)	< 0.001
ICU length of stay, days	1477	8 (3–18)	7 (3–16)	9 (5–18)	9 (4–20)	< 0.001
Hospital mortality, *n *(%)	1439	307 (21)	127 (17)	118 (22)	62 (40)	< 0.001
Hospital length of stay, days	1400	17 (11–28)	16 (10–27)	18 (12–29)	16 (11–27)	0.0039
Day 28 mortality, *n *(%)	1491	276 (19)	115 (15)	106 (19)	55 (35)	< 0.001
Day 60 mortality, *n *(%)	1491	308 (21)	130 (17)	117 (21)	61 (39)	< 0.001
Day 90 mortality, *n *(%)	1491	311 (21)	130 (17)	118 (21)	63 (40)	< 0.001

Results are expressed as *n* (%) or median (25th–75th percentiles)

*HFNC* high-flow nasal cannula, *NIV* noninvasive ventilation, *ICU* intensive care unit

**Table 3:** Factors associated with oxygenation failure by multivariate analysis among patients who were not intubated on the day of ICU admission

	Univariate OR (95% CI)	*P* value	Multivariate OR (95% CI)	*P* value
Age, years		< 0.001		0.078
≤ 75	–		–	
60–74	0.98 (0.72–1.32)		1.74 (0.93–3.28)	
40–59	0.58 (0.43–0.80)		1.33 (0.68–2.64)	
< 40	0.45 (0.27–0.74)		0.54 (0.14–1.82)	
Genre, woman	0.73 (0.58–0.92)	0.009	0.72 (0.44–1.19)	0.203
Body mass index, kg/m^2^	–	0.870	–	0.178
< 25				
25–29	1.05 (0.79–1.40)		0.89 (0.51–1.55)	
30–34	1.18 (0.86–1.63)		1.58 (0.83–3.01)	
35–39	1.14 (0.76–1.72)		1.01 (0.45–2.26)	
≥ 40	1.05 (0.60–1.85)		2.42 (0.73–8.26)	
Active smokers	1.00 (0.61–1.63)	0.996	0.83 (0.31–2.20)	0.701
SAPS II score	1.05 (1.04–1.06)	< 0.001	1.07 (1.04–1.09)	< 0.001
Treated hypertension	1.35 (1.10–1.66)	0.004	0.79 (0.51–1.23)	0.300
Known diabetes	1.31 (1.05–1.65)	0.019	0.74 (0.45–1.20)	0.222
Immunodepression^a^	1.43 (0.95–2.15)	0.085	0.85 (0.32–2.23)	0.747
Frailty score		0.009	–	0.875
1–3	–			
4	1.70 (1.16–2.51)		1.27 (0.51–3.32)	
5–9	1.46 (0.93–2.31)		1.07 (0.37–3.04)	
Delay between first signs and ICU admission, days		< 0.001	–	0.012
< 4	–			
4–7	0.84 (0.58–1.20)		0.74 (0.35–1.53)	
≥ 8	0.44 (0.31–0.62)		0.42 (0.20–0.87)	
**During the first 24 h in ICU**				
Oxygenation technique		0.019		0.013
Standard oxygen	–		–	
HFNC	0.98 (0.79–1.21)		0.60 (0.36–0.99)	
NIV	1.60 (1.13–2.27)		1.57 (0.78–3.21)	
Cardiovascular component of the SOFA score ≥ 3	3.37 (1.75–7.00)	< 0.001	1.45 (0.37–7.53)	0.608
Renal component of the SOFA score ≥ 3	1.06 (0.68–1.66)	0.785	0.35 (0.12–0.99)	0.048
Corticosteroids^b^	0.76 (0.55–1.05)	0.096	0.93 (0.46–1.88)	0.847
pH	0.12 (0.02–0.71)	0.010	0.95 (0.02–59.91)	0.980
PaCO_2_, mmHg	1.00 (1.00–1.00)	0.856	0.97 (0.94–1.00)	0.064
PaO_2_/FiO_2_^c^, mmHg		< 0.001	–	< 0.001
≤ 100	–			
101–200	0.43 (0.33–0.55)		0.32 (0.20–0.50)	
201–300	0.26 (0.17–0.37)		0.21 (0.10–0.41)	
> 300	0.25 (0.15–0.43)		0.23 (0.07–0.71)	
HCO3, mmol/L	0.97 (0.94–0.99)	0.006	1.00 (0.97–1.03)	0.993
Lactate, mmol/L	1.31 (1.12–1.57)	< 0.001	1.28 (1.06–1.67)	0.001
Lymphocyte count < 1 × 10^9^/L	1.59 (1.26–2.01)	< 0.001	1.20 (0.79–1.85)	0.394
Platelet count < 150 × 10^9^/L	1.76 (1.32–2.37)	< 0.001	1.16 (0.65–2.06)	0.621
Total bilirubin concentration, µmol/L	1.00 (0.99–1.01)	0.990	1.00 (0.97–1.02)	0.769
ICU admission period, March 29 to May 4, 2020	0.63 (0.51–0.77)	< 0.001	0.46 (0.29–0.72)	< 0.001

*OR* odds ratio, *CI* confidence interval, *HFNC* high-flow nasal cannula, *NIV* noninvasive ventilation, *SAPS* Simplified Acute Physiology Score, *SOFA* Sequential Organ Failure Assessment, *PaCO*_*2*_ partial pressure of carbon dioxide, *PaO*_*2*_*/FiO*_*2*_ partial pressure of oxygen to fraction of inspired oxygen ratio, *HCO3* bicarbonate

^a^Defined as hematological malignancies, active solid tumor, or having received specific anti-tumor treatment within a year, solid organ transplant, human immunodeficiency virus, or immunosuppressants

^b^Irrespective of the dose and the indication

^c^Calculated for all patients, including those on oxygen therapy by using conversion tables provided in the online supplement

### Factors associated with mortality and impact of HFNC and NIV on mortality

Overall 90-day mortality was 21%. Additional file 3: Table S3 shows the univariate analysis of the factors associated with 90-day mortality in patients who were not intubated on ICU admission. By multivariable analysis, factors associated with mortality were older age, known diabetes, severity as assessed by the SAPS II, higher frailty, shorter time between the first symptoms and ICU admission, lower PaO_2_/FiO_2_, and lower platelet count (Table 4). HFNC was not associated with a change in mortality (OR 0.90, 95% CI 0.61–1.33), but NIV was associated with an increased mortality (OR 2.75, 95% CI 1.79–4.21, *P* < 0.001).

**Table 4:** Factors associated with 90-day mortality by multivariate analysis among patients who were not intubated on the day of ICU admission

	*n*	Univariate HR (95% CI)	*P* value	Multivariate HR (95% CI)	*P* value
Age, years	1491		< 0.001		< 0.001
≤ 75		–		–	
60—74		0.43 (0.33–0.56)		0.60 (0.41–0.89)	
40—59		0.20 (0.14–0.27)		0.38 (0.23–0.63)	
< 40		0.17 (0.09–0.34)		0.33 (0.10–1.14)	
Genre, woman	1488	0.85 (0.65–1.10)	0.216	0.77 (0.52–1.15)	0.893
Body mass index, kg/m^2^	1370		0.254		0.072
< 25		–		–	
25—29		0.95 (0.70–1.27)		0.92 (0.61–1.41)	
30—34		0.74 (0.52–1.06)		0.79 (0.48–1.28)	
35—39		0.67 (0.41–1.08)		0.40 (0.18–0.86)	
≥ 40		0.97 (0.53–1.75)		1.01 (0.45–2.28)	
SAPS II score	1398	1.04 (1.03–1.05)	< 0.001	1.03 (1.01–1.04)	< 0.001
Treated hypertension	1485	1.45 (1.16–1.82)	< 0.001	0.78 (0.54–1.11)	0.844
Known diabetes	1486	1.76 (1.40–2.21)	< 0.001	1.28 (0.88–1.86)	0.015
Immunodepression^a^	1479	2.21 (1.58–3.10)	< 0.001	1.08 (0.60–1.92)	0.461
Frailty score	1379		< 0.001		< 0.001
1–3		–		–	
4		3.18 (2.34–4.33)		2.22 (1.39–3.54)	
5–9		4.23 (3.02–5.92)		1.67 (0.92–3.04)	
Delay between first signs and ICU admission, days	1424		< 0.001		< 0.001
< 4		–		–	
4–7		0.64 (0.47–0.88)		0.67 (0.43–1.05)	
≥ 8		0.41 (0.30–0.56)		0.42 (0.26–0.66)	
**During the first 24 h in ICU**					
Oxygenation technique	1491		< 0.001		< 0.001
Standard oxygen		–		–	
HFNC		1.27 (0.99–1.63)		0.90 (0.61–1.33)	
NIV		2.64 (1.95–3.56)		2.75 (1.79–4.21)	
Cardiovascular component of the SOFA score ≥ 3	1435	1.16 (0.64–2.12)	0.630	0.75 (0.32–1.75)	0.250
Renal component of the SOFA score ≥ 3	1416	2.43 (1.69–3.49)	< 0.001	1.81 (1.02–3.20)	0.099
PaO_2_/FiO_2_^b^, mmHg	1216		< 0.001		< 0.001
≤ 100		–		–	
101–200		0.68 (0.52–0.88)		0.58 (0.41–0.81)	
201–300		0.46 (0.29–0.72)		0.37 (0.20–0.68)	
> 300		0.24 (0.10–0.58)		0.08 (0.01–0.62)	
Lymphocyte count < 1 × 10^9^/L	1199	1.38 (1.06–1.80)	0.015	0.88 (0.62–1.24)	0.646
Platelet count < 150 × 10^9^/L	1342	2.16 (1.67–2.80)	< 0.001	2.02 (1.41–2.89)	< 0.001

*HR* hazard ratio, *CI* confidence interval, *HFNC* high-flow nasal cannula, *NIV* noninvasive ventilation, *SAPS* Simplified Acute Physiology Score, *SOFA* Sequential Organ Failure Assessment, *PaO*_*2*_*/FiO*_*2*_ partial pressure of oxygen to fraction of inspired oxygen ratio

^a^Defined as hematological malignancies, active solid tumor, or having received specific anti-tumor treatment within a year, solid organ transplant, human immunodeficiency virus, or immunosuppressants

^b^Calculated for all patients, including those on oxygen therapy by using conversion tables provided in the online supplement

The same analysis rerun after multiple imputations of missing data found similar results (see Additional file 4: Table S4).

Kaplan–Meier survival estimates according to oxygenation technique are provided in Fig. 2, Panel B, and showed increased mortality in the group initially treated with NIV.

## Discussion

In patients with acute respiratory failure due to COVID-19 and who were not intubated on the day of ICU admission, the results of our study showed, for each specific objective, the following: (1) HFNC and NIV, respectively, were used in 38% and 11% of patients, (2) HFNC but not NIV was independently associated with a reduction in oxygenation failure, and (3) HFNC was not associated with a reduction in 90-day mortality, and NIV was associated with increased 90-day mortality.

Despite international guidelines recommending early intubation of COVID-19 patients to protect healthcare workers [14] and despite experts recommending early intubation to prevent self-inflected lung injury in this patient population [15], HFNC and NIV were used increasingly during the first wave of the COVID-19 pandemic in France, Belgium, and Switzerland [8, 10, 16, 17]. The proportion of patients who received NIV was comparable to that reported by various COVID-19 cohort studies conducted in Europe and the USA [18–20]. It was also similar to the proportion of non-COVID-19 patients who received NIV in the LUNGSAFE cohort study [21]. On the other hand, the proportion of patients who received HFNC was higher in the present study than in these COVID and non-COVID cohorts. It is important to point that, in de novo acute hypoxemic respiratory failure, NIV is not recommended [6], while HFNC is now recommended [5].

The proportion of patients who were intubated was similar to that reported in other cohorts of COVID-19 patients [18, 22]. However, the impact of HFNC and NIV on oxygenation failure differed, with HFNC but not NIV being independently associated with a reduction in oxygenation failure. This benefit of HFNC has been reported in severe hypoxemic non-COVID-19 patients [4, 23], and more recently in COVID-19 patients [9, 18]. In de novo acute hypoxemic respiratory failure, the benefit of NIV is debated [6], and its use does not seem to be associated with a clear reduction in intubation rate in non-COVID-19 patients [6]. In COVID-19 patients, although retrospective studies have suggested a potential benefit of NIV on intubation [9, 18], a randomized control trial showed a reduction in intubation rate in patients receiving helmet NIV, but no improvement in the number of days free of respiratory support [22]. It is of note that we chose oxygenation failure (defined as intubation or death in the ICU without intubation) as the outcome rather than intubation alone as it is well established that HFNC and NIV may be given to do-not-intubate patients, as a ceiling therapy [24, 25]. This was the case in our cohort, where 4% patients died without being intubated, which is clearly a failure of oxygenation. Omitting these patients and choosing intubation as the sole outcome would have underestimated the oxygenation failure rate.

We did not find any impact of HFNC on mortality, while NIV was associated with increased mortality. In non-COVID-19 patients, there is not a clear benefit of either HFNC or NIV on mortality [4, 6]. In some patients, NIV could even be more deleterious than intubation [7]. One hypothesis is that a low expiratory tidal volume is almost impossible to achieve with NIV in patients with de novo acute hypoxemic respiratory failure [26], and a high tidal volume under NIV ((≥ 9 mL/kg of ideal body weight [26, 27]) could cause patient self-inflicted lung injury, which in turn may worsen the severity of lung disease [28]. Previously, a high tidal volume with NIV has been found to be independently associated with NIV failure [26, 27]. In COVID-19 patients, a retrospective study reported an association between NIV and mortality [18]; however, this association has not been observed in a randomized controlled trial that compared HFNC to helmet NIV [22]. In our patients treated with HFNC, the benefit of HFNC on oxygenation failure did not translate into a benefit in terms of mortality. A similar observation has been previously reported in a COVID-19 population [8]. It has also been suspected that HFNC could delay intubation excessively, resulting in more severe lung injury in patients intubated after HFNC failure [15]. However, this hypothesis has not been confirmed by a recent retrospective analysis, which showed similar mortality between patients intubated early after ICU admission and those intubated later [29].

The major strength of this study is the detailed report of clinical features, ventilatory management, and 90-day outcomes of a large, multicenter cohort of COVID-19 patients. We do, however, acknowledge several limitations to our study. First, the oxygenation technique was not randomized and it is possible that more severe patients were more likely to receive HFNC or NIV, precluding definitive conclusions due to be made. This includes the respective impact of HFNC and NIV on oxygenation failure and mortality. Second, the management of patients, and especially of oxygenation techniques and the decision of intubation, were not standardized. However, given the high number of patients, our cohort is likely to reflect the diversity of daily practices across centers. Third, we did not collect certain data that could be pertinent, such as awake prone positioning [30] and the ROX index [17, 31]. Fourth, some variables have missing data (as reported in the tables) due to the large number of patients included in a short period of time with intense clinical activity during the first outbreak of the pandemic. It is, however, of note that the sensitivity analysis with multiple imputations for missing data yielded similar results to the main analysis. Fifth, because the national health system was under extreme pressure with a need for a large number of ICU beds in some regions, we cannot be sure that policies of ICU admission and do-not-intubate decisions may have differed between regions and therefore between centers. Finally, we do not provide information on the contamination of caregivers, which could be higher with techniques that could generate a high level of aerosol transmission [9].

## Conclusions

Despite international recommendation and expert viewpoints to the contrary, HFNC and NIV were used increasingly during the first outbreak of the COVID-19 pandemics in France, Belgium, and Switzerland. Although HFNC was not associated with a reduction in 90-day mortality, it was associated with less oxygenation failure. This study highlights that HFNC can be useful and as safe as standard oxygen in a large cohort of COVID-19 patients and paves the way for future randomized control trials investigating the benefit of HFNC in these patients. On the contrary, NIV was associated with increased mortality. This observed increased mortality of NIV, if sustained in a prospective randomized trial, would support not using it in COVID-19 patients.

## Supplementary Information

**Additional file 1**. **Table S1.** Estimating inspired fraction of oxygen (FiO_2_) from a given oxygen flow.

**Additional file 2**. **Table S2.** Univariate analysis: factors associated with oxygenation failure among patients who were not intubated on the day of intensive care unit (ICU) admission.

**Additional file 3**. **Table S3.** Univariate analysis: factors associated with 90-days mortality among patients who were not intubated on the day of intensive care unit (ICU) admission.

**Additional file 4**. **Table S4.** Factors associated with 90-days mortality by multivariate analysis among patients who were not intubated on the day of intensive care unit (ICU) admission after multiple imputation for missing data.

## Data Availability

The datasets used and/or analyzed during the current study are available from the corresponding author on reasonable request.

## Abbreviations

ICU: Intensive care unit
HFNC: High-flow nasal cannula
NIV: Noninvasive ventilation
COVID-19: Coronavirus disease 2019
SARS-CoV-2: Severe acute respiratory syndrome coronavirus 2
PaO_2_/FiO_2_: Pressure of arterial oxygen to fraction of inspired oxygen concentration
