# Supplementary material for: Benefits and risks of noninvasive oxygenation strategy in COVID-19: a multicenter, prospective cohort study (COVID-ICU) in 137 hospitals
Source: Crit Care. 2021 Dec 8;25:421. doi: 10.1186/s13054-021-03784-2 (PMC8653629; doi:10.1186/s13054-021-03784-2)
Supplement: Supplementary file 1 — Additional file 1. Table S1. Estimating inspired fraction of oxygen (FiO2) from a given oxygen flow. [file 13054_2021_3784_MOESM1_ESM.docx]

**Table S1. Estimating inspired fraction of oxygen (FiO_2_) from a given oxygen flow**

| **Oxygenation technique** | **Oxygen flow (l/min)** | **Estimated FiO_2_ (%)** |
| --- | --- | --- |
| ***Nasal cannulae*** | 1 | 24 |
|  | 2 | 28 |
|  | 3 | 32 |
|  | 4 | 36 |
|  | 5 | 40 |
|  | 6 | 44 |
| ***Nasopharyngeal catheter*** | 4 | 40 |
|  | 5 | 50 |
|  | 6 | 60 |
| ***Face mask*** | 5 | 40 |
|  | 6-7 | 50 |
|  | 7-8 | 60 |
| ***Face mask with reservoir*** | 6 | 60 |
|  | 7 | 70 |
|  | 8 | 80 |
|  | 9 | 90 |
|  | 10 | 95 |
