# Supplementary material for: Benefits and risks of noninvasive oxygenation strategy in COVID-19: a multicenter, prospective cohort study (COVID-ICU) in 137 hospitals
Source: Crit Care. 2021 Dec 8;25:421. doi: 10.1186/s13054-021-03784-2 (PMC8653629; doi:10.1186/s13054-021-03784-2)
Supplement: Supplementary file 2 — Additional file 2. Table S2. Univariate analysis: factors associated with oxygenation failure among patients who were not intubated on the day of intensive care unit (ICU) admission. [file 13054_2021_3784_MOESM2_ESM.docx]

**Table S2. Univariate analysis: factors associated with oxygenation failure among patients who were not intubated on the day of intensive care unit (ICU) admission**

|  | **No.** | **All**  **patients**  **(n=1491)** | **Oxygenation success**  **(n=752)** | **Oxygenation failure**  **(n=739)** | **P value** |
| --- | --- | --- | --- | --- | --- |
| Age, *years,* | 1491 | 63 (54–71) | 61 (50–71) | 64 (56–72) | <0.001 |
| Women, *n (%)* | 1488 | 397 (27) | 223 (29) | 174 (24) | 0.008 |
| Body mass index, *kg/m^2^* | 1370 | 28 (25–31) | 28 (25–31) | 28 (25–31) | 0.466 |
| ≥30 kg/m^2^, *n (%)* | 1370 | 504 (37) | 240 (36) | 264 (38) | 0.330 |
| Active smokers, *n (%)* | 1465 | 67 (5) | 34 (4) | 33 (5) | 0.996 |
| SAPS II score | 1398 | 30 (23–37) | 27 (22–34) | 33 (26–42) | <0.001 |
| SOFA score at ICU admission | 1290 | 3 (2–4) | 3 (2–4) | 3 (2–5) | <0.001 |
| Treated hypertension, *n (%)* | 1485 | 687 (46) | 319 (43) | 368 (50) | 0.004 |
| Known diabetes, *n (%)* | 1486 | 409 (28) | 186 (25) | 223 (30) | 0.019 |
| Immunodeficiency^a^, *n (%)* | 1479 | 102 (7) | 43 (6) | 59 (8) | 0.086 |
| Clinical frailty scale | 1379 | 2 (2–3) | 2 (1–3) | 2 (2–3) | 0.001 |
| Time between first symptoms and ICU admission, *days* | 1435 | 9 (6-12) | 10 (7-13) | 8 (6-11) | <0.001 |
| **During the first 24 hours in ICU** |  |  |  |  |  |
| Oxygenation technique | 1491 |  |  |  | 0.019 |
| Standard oxygen, n (%) |  | 766 (51) | 394 (52) | 372 (50) |  |
| HFNC, n (%) |  | 567 (38) | 295 (39) | 272 (37) |  |
| NIV, n (%) |  | 158 (11) | 63 (8) | 95 (12) |  |
| Hemodynamic component of the SOFA | 1435 | 0 (0–0) | 0 (0–0) | 0 (0–0) | 0.297 |
| Renal component of the SOFA | 1416 | 0 (0–0) | 0 (0–0) | 0 (0–0) | 0.015 |
| Corticosteroids^b^, *n (%)* | 1476 | 165 (11) | 93 (13) | 72 (10) | 0.097 |
| Blood gases |  |  |  |  |  |
| pH | 1365 | 7.46 (7.42-7.48) | 7.46 (7.43-7.48) | 7.45 (7.42-7.48) | 0.017 |
| PaCO_2_, *mmHg* | 1367 | 36 (32–39.5) | 36 (33–40) | 36 (32–39) | 0.035 |
| PaO_2_/FiO_2_^c^ | 1216 | 122 (82–177) | 140 (96–206) | 101 (72–147) | <0.001 |
| HCO_3_, *mmol/L* | 1357 | 25 (23–27) | 25 (23–27) | 25 (22–27) | <0.001 |
| Lactate, *mmol/L* | 1281 | 1.1 (0.9–1.5) | 1.1 (0.9–1.4) | 1.2 (1–1.6) | <0.001 |
| Biology |  |  |  |  |  |
| Lymphocyte count, *× 10⁹/L* | 1199 | 0.8 (0.6–1.2) | 0.9 (0.6–1.3) | 0.8 (0.5–1.1) | <0.001 |
| Platelet count, *× 10⁹/L* | 1342 | 222 (167–289) | 239 (180–307) | 200 (159–270) | <0.001 |
| Total bilirubin, *µmol/L* | 1023 | 9 (7–12) | 9 (7–12.9) | 9 (7–12) | 0.832 |
| Serum creatinine, *µmol/L* | 1369 | 71 (59–94) | 69 (57–87) | 75 (61–101) | <0.001 |
| D–dimers, *µg/L* | 548 | 1159 (647–2204) | 1144 (625–2330) | 1173 (713–2070) | 0.820 |
| **Period of admission** | 1491 |  |  |  | <0.001 |
| 25 February to 28 March, 2020, *n (%)* |  | 713 (48) | 317 (42) | 396 (54) |  |
| 29 March to 4 May 2020, *n (%)* |  | 778 (52) | 435 (58) | 343 (46) |  |

Abbreviations: HFNC, high flow nasal cannula; NIV, non–invasive ventilation; SAPS, simplified acute physiology score; SOFA, Sequential Organ Failure Assessment; PaCO_2_, partial pressure of carbon dioxide; PaO_2_/FiO_2_, partial pressure of oxygen to fraction of inspired oxygen ratio; HCO3, bicarbonate.

Results are expressed as n (%) or median (25^th^ – 75^th^ percentiles).

^a^ defined as hematological malignancies, active solid tumor, or having received specific anti–tumor treatment within a year, solid–organ transplant, human immunodeficiency ,virus, or immunosuppressants.

^b^ irrespective of the dose and the indication

^c^ calculated for all patients, including those on oxygen therapy by using conversion tables provided in the online supplement
