# Supplementary material for: Benefits and risks of noninvasive oxygenation strategy in COVID-19: a multicenter, prospective cohort study (COVID-ICU) in 137 hospitals
Source: Crit Care. 2021 Dec 8;25:421. doi: 10.1186/s13054-021-03784-2 (PMC8653629; doi:10.1186/s13054-021-03784-2)
Supplement: Supplementary file 4 — Additional file 4. Table S4. Factors associated with 90-days mortality by multivariate analysis among patients who were not intubated on the day of intensive care unit (ICU) admission after multiple imputation for missing data. [file 13054_2021_3784_MOESM4_ESM.docx]

**Table S4. Factors associated with 90-days mortality by multivariate analysis among patients who were not intubated on the day of intensive care unit (ICU) admission after multiple imputation for missing data**

|  | **Multivariate HR (95% CI)** | **P value** |
| --- | --- | --- |
| Age, *years* |  | <0.001 |
| ≤75 | - |  |
| 60 - 74 | 0.57 (0.44 - 0.74) |  |
| 40 - 59 | 0.36 (0.25 - 0.53) |  |
| <40 | 0.53 (0.26 - 1.1) |  |
| Genre, *woman* | 0.76 (0.57 - 1.02) | 0.068 |
| Body mass index, *kg/m^2^* |  | 0.442 |
| <25 | - |  |
| 25 - 29 | 1.06 (0.79 - 1.44) |  |
| 30 - 34 | 0.79 (0.54 - 1.15) |  |
| 35 - 39 | 0.77 (0.47 - 1.26) |  |
| ≥40 | 0.84 (0.43 - 1.62) |  |
| SAPS II score | 1.02 (1.01 - 1.03) | <0.001 |
| Treated hypertension | 0.9 (0.7 - 1.16) | 0.441 |
| Known diabetes | 1.17 (0.89 - 1.54) | 0.255 |
| Immunodepression^a^ | 1.43 (0.98 - 2.1) | 0.067 |
| Frailty score |  | <0.001 |
| 1-3 | - |  |
| 4 | 1.92 (1.33 - 2.78) |  |
| 5-9 | 2.52 (1.7 - 3.74) |  |
| Delay between first signs and ICU admission, *days* |  | 0.001 |
| < 4 | - |  |
| 4 – 7 | 0.78 (0.52 - 1.17) |  |
| ≥8 | 0.53 (0.36 - 0.79) |  |
| **During the first 24 hours in ICU** |  |  |
| Oxygenation technique |  | <0.001 |
| Standard oxygen | - |  |
| HFNC | 1.06 (0.8 - 1.41) |  |
| NIV | 2.22 (1.6 - 3.06) |  |
| Cardiovascular component of the SOFA score ≥ 3 | 0.96 (0.52 - 1.78) | 0.874 |
| Renal component of the SOFA score ≥ 3 | 1.64 (1.07 - 2.52) | 0.025 |
| PaO_2_/FiO_2_^b^, *mmHg* |  | 0.004 |
| ≤100 | - |  |
| 101 – 200 | 0.68 (0.51 - 0.92) |  |
| 201 – 300 | 0.47 (0.25 - 0.87) |  |
| >300 | 0.22 (0.09 - 0.55) |  |
| Lymphocyte count <1x10^9^/L | 1 (0.76 - 1.31) | 0.711 |
| Platelet count <150x10^9^/L | 1.82 (1.38 - 2.39) | <0.001 |

Abbreviations: HR, hazard ratio; CI, confidence interval; HFNC, high flow nasal cannula; NIV, non–invasive ventilation; SAPS, simplified acute physiology score; SOFA, Sequential Organ Failure Assessment; PaO_2_/FiO_2_, partial pressure of oxygen to fraction of inspired oxygen ratio.

^a^ defined as hematological malignancies, active solid tumor, or having received specific anti–tumor treatment within a year, solid–organ transplant, human immunodeficiency ,virus, or immunosuppressants.

^b^ calculated for all patients, including those on oxygen therapy by using conversion tables provided in the online supplement
